# Chromosome divergence during evolution of the tetraploid clawed frogs, *Xenopus mellotropicalis* and *Xenopus epitropicalis* as revealed by Zoo-FISH

**DOI:** 10.1371/journal.pone.0177087

**Published:** 2017-05-18

**Authors:** Martin Knytl, Ondřej Smolík, Svatava Kubíčková, Tereza Tlapáková, Ben J. Evans, Vladimír Krylov

**Affiliations:** 1 Department of Cell Biology, Faculty of Science, Charles University, Prague, Czech Republic; 2 Department of Genetics and Reproduction, CEITEC -Veterinary Research Institute, Brno, Czech Republic; 3 Department of Biology, Life Sciences Building Room 328, Mc Master University, Hamilton, Ontario, Canada; Virginia Tech, UNITED STATES

## Abstract

Whole genome duplication (WGD) generates new species and genomic redundancy. In African clawed frogs of the genus *Xenopus*, this phenomenon has been especially important in that (i) all but one extant species are polyploid and (ii) whole genome sequences of some species provide an evidence for genomic rearrangements prior to or after WGD. Within *Xenopus* in the subgenus *Silurana*, at least one allotetraploidization event gave rise to three extant tetraploid (2*n* = 4*x* = 40) species–*Xenopus mellotropicalis*, *X*. *epitropicalis*, and *X*. *calcaratus–*but it is not yet clear the degree to which these tetraploid genomes experienced rearrangements prior to or after allotetraploidization. To explore genome evolution during diversification of these species, we performed cytogenetic analyses of *X*. *mellotropicalis*, including assessment of the localization of nucleolar organizer region, chromosome banding, and determination of the *p*/*q* arm ratios for each chromosome pair. We compared these data to a previously characterized karyotype of *X*. *epitropicalis*. Morphometric, C-banding and Zoo-FISH data support a previously hypothesized common allotetraploid predecessor of these species. Zoo-FISH with whole chromosome painting (WCP) probes derived from the closely related diploid species *X*. *tropicalis* confirmed the existence of ten chromosomal quartets in *X*. *mellotropicalis* somatic cells, as expected by its ploidy level and tetraploid ancestry. The *p*/*q* arm ratio of chromosome 2a was found to be substantially different between *X*. *mellotropicalis* (0.81) and *X*. *epitropicalis* (0.67), but no substantial difference between these two species was detected in this ratio for the homoeologous chromosome pair 2b, or for other chromosome pairs. Additionally, we identified variation between these two species in the locations of a heterochromatic block on chromosome pair 2a. These results are consistent with a dynamic history of genomic rearrangements before and/or after genome duplication, a surprising finding given the otherwise relatively conserved genomic structure of most frogs.

## Introduction

Whole genome duplication (WGD) is an important evolutionary phenomenon that occurs in animals, plants, and several other organisms, and can be associated with chromosomal rearrangements [1–4]. WGD takes place either by allopolyploidization, which is associated with interspecies hybridization, or by autopolyploidization, in which WGD occurs within a single ancestral species [5]. Polyploidization is frequently followed by instantaneous or eventual diploidization of the genome [6], such that during cell division, bivalents rather than multivalents form [7,8].

The African clawed frog genus *Xenopus* [9] includes subgenera *Silurana* [10] and *Xenopus*, and contains species with several ploidy levels (diploid, tetraploid, octoploid, or dodecaploid) [11–15]. African clawed frogs reproduce sexually, and meiosis is characterized by the formation of bivalents, the occurence of crossing-over, and the production of reduced gametes [16]. The genus *Xenopus* includes 29 species, 28 of which are polyploid, and whose evolutionary relationships bifurcate due to speciation without polyploidization and also reticulate due to speciation by allopolyploidization [17]. The subgenus *Silurana* comprises *Xenopus tropicalis* [10], the only known extant diploid *Xenopus* species (2*n* = 2*x* = 20, where *n* refers to the number of chromosomes in a gamete of the extant species, and *x* refers to the number of chromosomes in a gamete of the most recent diploid ancestor of the extant species), and three tetraploid species with 40 chromosomes (2*n* = 4*x* = 40): *X*. *epitropicalis* [18], *X*. *mellotropicalis*, and *X*. *calcaratus* [17]. Phylogenetic studies indicate that WGD in *Silurana* took place by allopolyploidization rather than autopolyploidization [16,17,19]. Subgenus *Xenopus* is represented by 25 described species, including allotetraploids, allooctoploids, and allododecaploids, which have 36, 72 or 108 chromosomes, respectively [13,14]. Diploid species within the subgenus *Xenopus* are unknown (and perhaps extinct) and presumed to have had 2*n* = 2*x* = 18 chromosomes [20]. Octoploid and dodecaploid species in the subgenus *Xenopus* arose via at least six independent polyploidization events [17].

Karyotypes of *X*. *tropicalis*, *X*. *epitropicalis* and *X*. *mellotropicalis* have been previously analyzed. *X*. *mellotropicalis* was previously known as a *X*. new tetraploid 1 [21,22] or *X*. species nova VII [16]. These studies indicate the presence of two types of secondary constrictions, which are regions of the chromosome that appear as a constriction apart from the region associated with the centromere (which is the primary constriction) [11]. One secondary constriction that was detected by silver nitrate staining, is associated with nucleolar organizer regions (NORs) on *X*. *tropicalis* chromosome pair (XTR) 7 (i.e. XTR 7) and *X*. *epitropicalis* 7a (XEP 7a). Another secondary constriction, which was revealed by C-banding, is present on *X*. *tropicalis* chromosome 9 but lacking in *X*. *epitropicalis* [23] and *X*. *mellotropicalis* [16]. This type of secondary constrictiction has been called non-specific [16]. High resolution banding patterns of all three species were performed for each chromosome using 5-bromodeoxyuridine (BrdU) and deoxythimidine (dT). Very late-replicating C- and G-bands were also observed [22]. In several *Xenopus* species each characterized by a different ploidy level, Tymowska [16] observed the localization of only two NORs on one homologous chromosome pair. In a pair of *X*. *borealis* [24] individuals from the region near Samburu, in Kenya, Jotterand and Fischberg [25] detected a balanced reciprocal translocation event between the *p* arm of 7a and the *p* arm of 4b (bearing NOR) that was associated with either one or three nucleolar organizer regions in offspring karyotypes instead of two.

Chromosome evolution in *Xenopus* was recently investigated using fluorescence *in situ* hybridization (FISH) employing chromosome painting probes [26] and a comparative cytogenetic map was constructed using 198 physically localized genes [27,28]. A probe generated from laser microdissected *X*. *tropicalis* chromosomes labelled chromosomal quartets (consisting of two homoeologous pairs of homologous chromosomes) in *X*. *laevis* [29], except for a painting probe derived from the smallest XTR 10, which had a dispersed signal indicating independent fusions between XTR 10, XTR 9 and XTR 10, XTR 8 respectively in diploid ancestors. Results supported allotetraploid origin of *X*. *laevis* and the prior existence of two diploid ancestral species with 18 chromosomes [26], the descendants of which may be extinct. Only fusion between XTR 9 and XTR 10 was further evidenced by genome sequences [27,28], including the identification of telomere sequences at the fusion junction. Within the subgenus *Silurana*, an allotetraploidization origin of three species was hypothesized based on phylogenetic relationships among homoeologous copies of linked genes *RAG1* and *RAG2* [30,31].

To further understand genome evolution in African clawed frogs, we performed cytogenetic comparative analysis of *X*. *mellotropicalis* and *X*. *tropicalis* using chromosome banding, FISH with ribosomal 5S and 28S probes and cross-species fluorescence *in situ* hybridization (Zoo-FISH) with WCP probes derived from microdissected *X*. *tropicalis* chromosomes. We additionally provide an evolutionary interpretation of our results, that incorporates information from previous cytogenetic analyses of *X*. *epitropicalis*.

## Materials and methods

### Ethics statement

This study was carried out in accordance with Act No. 246/1992 Coll., on the protection of animals against cruelty, as amended by Act No 162/1993 Coll., Act No 193/1994 Coll., Act No 243/1997 Coll., finding of the Constitutional Court No 30/1998 Coll., Act No 77/2004 Coll., Act No 413/2005 Coll., Act No 77/2006 Coll. and Act No 312/2008 Coll. An official permission was issued to the Faculty of Science, Charles University by the Ministry of Education, Youth and Sports of the Czech Republic (No. MSMT-37376/2014-4, expiry date 3. 3. 2019).

### Primary cell culture

*X*. *mellotropicalis* animals originated from Gabon and were raised at McMaster University, Canada. *X*. *tropicalis* animals originating from Ivory Coast were raised at the Faculty of Science Charles University, Prague, Czech Republic. The *X*. *tropicalis* and the *X*. *mellotropicalis* primary cell cultures were established from the hind limbs of 10 tadpoles at stage NF55 (±1) as previously described by Sinzelle et al. [32]. The tadpoles were anesthetized by incubation for 5 minutes in 0.4% MS-222 (Sigma-Aldrich, St. Louis, MO, USA) and then washed extensively with sterile MilliQ water following death. The hind limbs were dissected and homogenized in cultivation medium consisting of 33.3% L-15 and 33.3% RPMI 1640 HEPES modification medium (both Sigma-Aldrich) supplemented with 10% FBS (Thermo Fisher Scientific, Waltham, MA, USA), 1.33 mg/ml sodium bicarbonate, 2 mM L-glutamine, 1 mM sodium pyruvate and 50 μg/ml gentamicin (all Sigma-Aldrich). The explants were then cultivated at 29.5°C with 5.5% CO_2_ for 5 days without disturbance, and undissociated portions of tissue were then removed every 3 days during medium changing. A first passage was performed after two-week cultivation with a low concentration of trypsin-EDTA solution (0.25% trypsin-0.1% EDTA, Sigma-Aldrich). Following this, the concentration of the trypsin-EDTA solution was increased in subsequent passages to a concentration of 0.5% trypsin-0.2% EDTA. For cryopreservation, cell aliquots were stored in LN_2_ in the cultivation medium with the addition of 10% DMSO. Prepared cell cultures exhibited a homogenous epithelioid morphology.

### Chromosome banding

Sequential chromosome banding (4’, 6-diamidino-2-phenylindole—DAPI; Chromomycin A_3_ –CMA_3_, Sigma-Aldrich; C-banding) with destaining and cleaning steps was performed on *X*. *tropicalis* and *X*. *mellotropicalis* (XME) chromosomes. The chromosome banding protocols consisted of fluorescent CMA_3_/DAPI banding, including destaining procedures according to Rábová et al. [33], with the following minor modifications: slides were stained by CMA_3_ solution for 60 instead of 15 minutes (min) at room temperature (RT) with subsequent dehydration through ethanol series (70, 80 and 96% for 2 min each) and final C-banding/DAPI protocol included slide aging for 60 min at 60°C. Chromosomes were then denatured in a 5.3% Ba(OH)_2_ solution for 3 min at 45°C and incubated in 1x SSC for 90 min at 65°C. DAPI combined with Vectashield (Cytocell, Cambridge, United Kingdom) was used for chromosome counterstaining.

### Preparation of 5S and 28S ribosomal DNA probes and FISH

The visualization of 5S and 28S rDNA loci on *X*. *tropicalis* and *X*. *mellotropicalis* chromosomes was performed using double colour FISH. Probes were generated from genomic DNA by PCR amplifiying either 5S or 28S. The sequences of 5S primers were slightly modified: 5’-CAGGCTGGTATGGCCGTAAGC-3’ and 5’-TACGCTGGTATGGCCGTAAGC-3’ [34]; and those of the 28S primers were: 5’-AAACTCTGGTGGAGGTCCGT-3’ and 5’-CTTACCAAAAGTGGCCCACTA-3’ [35]. The temperature profile for the amplification of 5S locus was as follows: initial denaturation step for 5 min at 95°C, followed by 34 cycles (95°C for 15 sec, 55°C for 30 sec and 72°C for 30 sec) with final extension step at 72°C for 5 min. Conditions for PCR of 28S locus were as follows: initial denaturation step for 3 min at 94°C, followed by 33 cycles (94°C for 30 sec, 53°C for 30 sec and 72°C for 45 sec) with final extension step at 72°C for 10 min. The 5S PCR product labelled by the Bio-16-dUTP (Roche, Mannheim, Germany) was detected by CY^™^3-Streptavidin (Invitrogen, Camarillo, CA, USA) with 10% GNS/PBS. The 28S PCR product labelled by the Dig-11-dUTP was detected by Anti-digoxigenin-Fluorescein (Roche) with 0.5% BSA/PBS. Both PCR amplicons were purified using a GeneJET^™^ Gel Extraction Kit (Thermo Fisher Scientific). In total 44 μl of the hybridization mixture containing 250 ng of either 5S or 28S PCR products, 50% deionized formamide, 2x SSC, 10% dextran sulphate and water was placed on a slide and covered with a 24 x 50 mm coverslip. Hybridization, post-hybridization washing, and visualization of 5S and 28S rDNA signals were carried out as described in the section on painting FISH.

### Preparation of *X*. *tropicalis* WCP probes

*X*. *tropicalis* metaphase spreads were prepared from cell cultures as previously described by Krylov et al. [26]. Chromosomes were identified by means of their relative length and short/long (*p*/*q*) arm ratio and using the chromosomal nomenclature for *X*. *tropicalis* defined by Khokha et al. [36]. Individual chromosomes were isolated by laser microdissection as previously described in Kubickova et al. [37] and Seifertova et al. [38]. The preparation of WCP probes was conducted as described by Krylov et al. [26].

### Painting FISH and Zoo-FISH analysis

Hybridization of painting probes, stringency washing and signal visualization were carried out as described by Krylov et al. [26] with minor changes and with supplementation of painting probes from XTR 2 and XTR 4. We used autoclaved *X*. *tropicalis* genomic DNA as a competitor (blocking DNA) according to Bi and Bogart [39]. After denaturation, the probe was reannealed for 90 min at 37°C. The hybridization mixture was incubated with chromosomes for 48 hours at 37°C in a wet chamber. The protocol for Zoo-FISH experiments was similar as for painting FISH but with minor changes, including more probe (750 ng), a longer hybridization time (72 hours), a lower temperature during posthybridization washing in 50% formamide (38°C), and the renaturation step was done for 120 instead of 90 min.

## Results

### Chromosome banding and morphometric analysis

**Fig 1:**
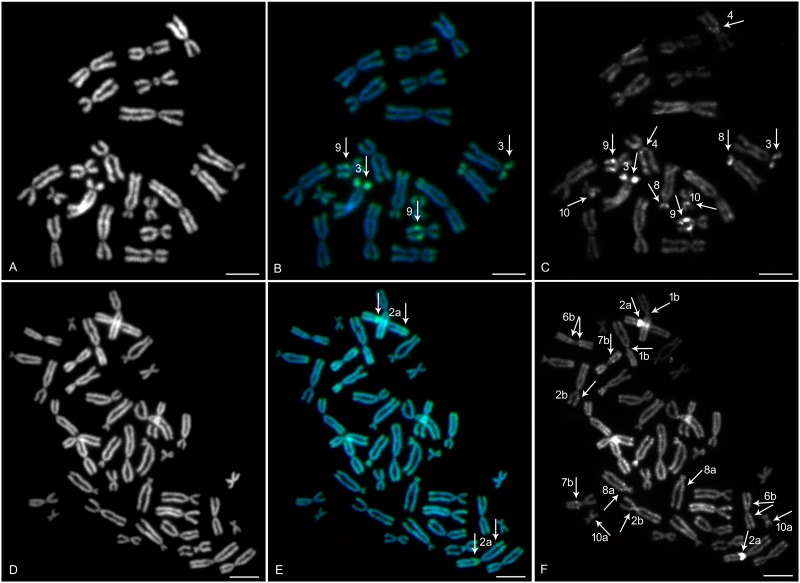
Sequential fluorescent chromosome banding on metaphase spread of *X*. *tropicalis* and *X*. *mellotropicalis*. DAPI (B&W) counter-stained metaphase spreads showed all (A) 20 *X*. *tropicalis* and/or (D) 40 *X*. *mellotropicalis* chromosomes. (B) CMA_3_ (green) and (C) C-banding (B&W) in *X*. *tropicalis* stained the part of *q* arms of XTR 9 and *p* arms of XTR 3. Moreover, (C) the pericentric region of XTR 4 and 10 and *p* arms of XTR 8 were weakly stained by C-banding. *X*. *mellotropicalis* chromosomes stained by (E) CMA_3_ (green) and (F) C-banding (B&W) revealed positive bands located on the *p* arm pericentric region of XME 2a. In addition, (F) C-banding detected further minor heterochromatic blocks on *X*. *mellotropicalis* chromosomes 1b, 2b, 6b, 7b, 8a and 10a. (B, C, E, F) All arrows show heterochromatic blocks. Scale bar represents 10 μm.

In *X*. *mellotropicalis* and *X*. *tropicalis*, sequential fluorescent chromosome banding–(i) CMA_3_/DAPI, (ii) C-banding/DAPI identified homologous chromosome pairs, and DAPI produced strong signals on each chromosome, including discernable differences in chromosome morphology (Fig 1). CMA_3_-positive heterochromatic bands were identified on the *q* arms of XTR 9, *p* arms of XTR 3 (Fig 1B), and on the *p* + *q* arms of XME 2a (Fig 1E). All *X*. *tropicalis* and *X*. *mellotropicalis* chromosomes bore weak bands on telomeres, that presumably were caused by repetitive sequences. C-banded regions showed blocks of constitutive heterochromatin—which is generally composed of repetitive sequences—that co-localized with CMA_3_-positive bands (Fig 1C and 1F). Pericentric regions of XTR 4 and 10 and *p* arms of XTR 8 were also labelled (Fig 1C). In addition to a strongly stained heterochromatic block on XME 2a, C-banding in *X*. *mellotropicalis* exhibited a faint signal in portions of stained regions on chromosomes 1b, 2b, 6b, 7b, 8a and 10a. In general, C-positive heterochromatic block patterns were consistent within particular homologous pairs of *X*. *tropicalis* and *X*. *mellotropicalis*, but exhibited some differences between homoeologous pairs of *X*. *mellotropicalis*.

All analyzed specimens of *X*. *mellotropicalis* possessed 40 chromosomes in their karyotypes. In a comparison of the *p*/*q* arm ratios of *X*. *mellotropicalis* (33 metaphases) and a previously described karyotype of *X*. *epitropicalis* [23], the largest difference was in chromosomes 2a where the ratio for *X*. *mellotropicalis* was 0.81 and the ratio for *X*. *epitropicalis* was 0.67 (Table 1).

**Table 1:** Comparison of the *p*/*q* arm ratio among *X*. *tropicalis*, *X*. *mellotropicalis* and *X*. *epitropicalis* chromosomes.

Chromosome	*X*. *tropicalis p*/*q* arm ratio [36]	*X*. *mellotropicalis* (this study)		*X*. *epitropicalis* [23]	
		Homoeolog	*p*/*q* arm ratio	Homoeolog	*p*/*q* arm ratio
**1**	**0,73**	**a**	**0,70**	**a**	**0,71**
		**b**	**0,67**	**b**	**0,70**
**2**	**0,63**	**a**	**0,81**	**a**	**0,67**
		**b**	**0,62**	**b**	**0,68**
**3**	**0,31**	**a**	**0,20**	**a**	**0,21**
		**b**	**0,20**	**b**	**0,18**
**4**	**0,58**	**a**	**0,60**	**a**	**0,64**
		**b**	**0,62**	**b**	**0,67**
**5**	**0,60**	**a**	**0,42**	**a**	**0,49**
		**b**	**0,41**	**b**	**0,44**
**6**	**0,89**	**a**	**0,88**	**a**	**0,89**
		**b**	**0,88**	**b**	**0,88**
**7**	**0,72**	**a**	**0,66**	**a**	**0,72**
		**b**	**0,79**	**b**	**0,78**
**8**	**0,22**	**a**	**0,21**	**a**	**0,28**
		**b**	**0,21**	**b**	**0,23**
**9**	**0,68**	**a**	**0,88**	**a**	**0,80**
		**b**	**0,72**	**b**	**0,80**
**10**	**0,70**	**a**	**0,70**	**a**	**0,76**
		**b**	**0,72**	**b**	**0,75**

Different *p*/*q* arm ratio values between *X*. *tropicalis* chromosome 2 and homoeologous counterparts 2a in *X*. *mellotropicalis* and *X*. *epitropicalis* are highlighted in blue.

### 5S and 28S rDNA FISH

**Fig 2:**
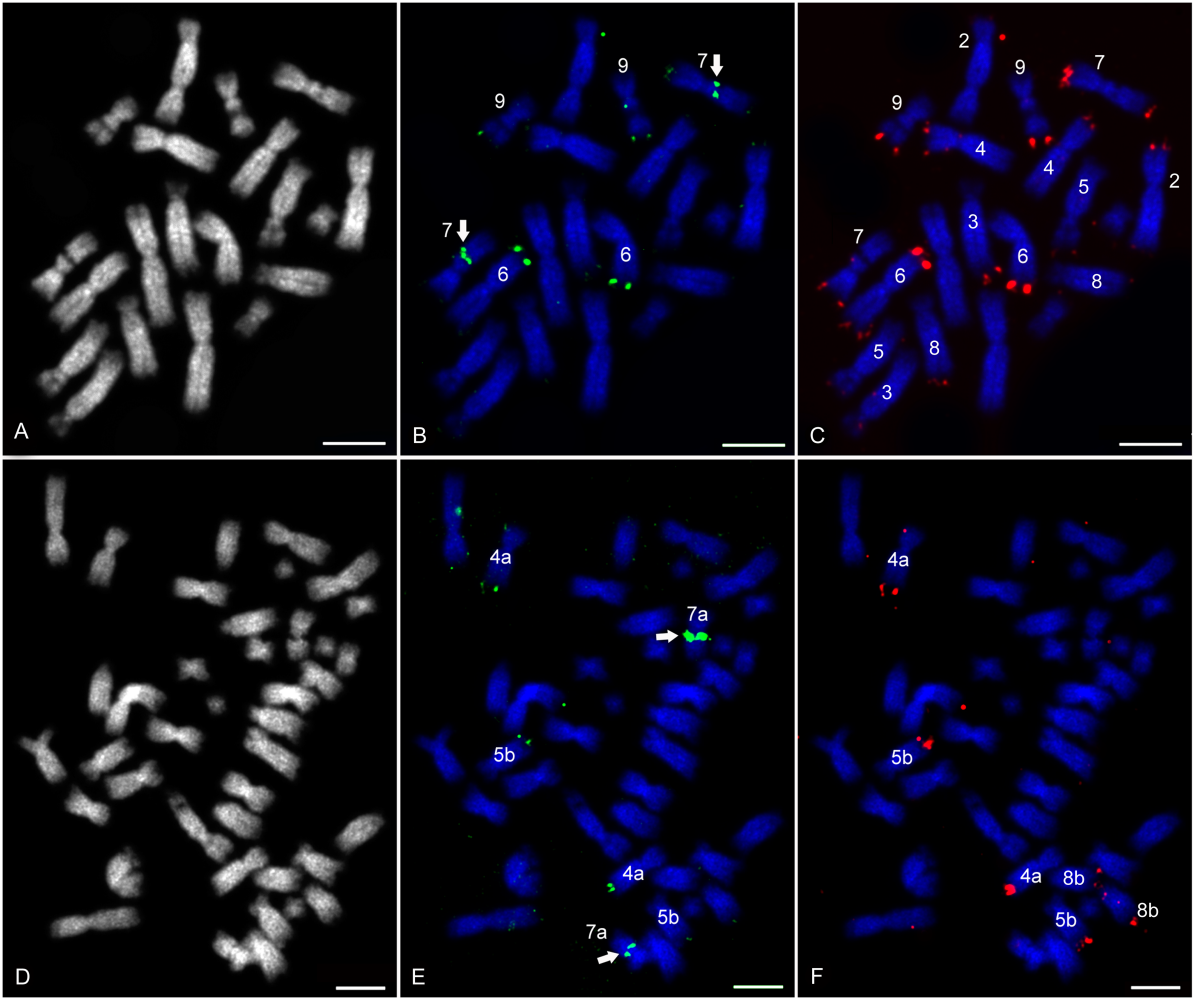
Double colour 5S and 28S rDNA FISH on *X*. *tropicalis* and *X*. *mellotropicalis* chromosomes. DAPI counter-stained metaphase spreads showed all (A) 20 chromosomes (B&W) in *X*. *tropicalis* and/or (D) 40 chromosomes in *X*. *mellotropicalis*. 28S rDNA amplicon labelled by Digoxigenin-11-dUTP (green) stained (B) XTR 7 secondary constriction and telomeres of XTR 6, 7 and 9 and/or (E) XME 7a secondary constriction and telomeres of XME 4a and 5b. 5S rDNA amplicon labelled by Biotin-16-dUTP (red) revealed positive signals on telomeric segments of (C) XTR 2, 3, 4, 5, 6, 7, 8 and 9 and/or (F) XME 4a, 5b and 8b. Arrows show NORs situated on secondary constrictions of (B) XTR 7 and (E) XME 7a. Scale bar represents 10 μm.

The first double colour FISH was performed on *Xenopus* chromosomes with digoxigenin and biotin labelled probes. The number of NORs detected by 28S rDNA FISH was the same in *X*. *tropicalis*, a diploid species, and in *X*. *mellotropicalis*, which evolved from a tetraploid ancestor although its genome is disomic now. The 28S probe detected two NORs situated on the nucleolar secondary constriction of XTR 7 *q* arms (Fig 2B) and XME 7a (Fig 2E). Telomeric 28S rDNA signals were located on XTR 6, 7 and 9 (Fig 2B) and on XME 4a and 5b (Fig 2E). All 5S rDNA loci were situated on telomeres of XTR 2, 3, 4, 5, 6, 7, 8, 9 (Fig 2C) and XME 4a, 5b and 8b (Fig 2F). Some of 5S rDNA sites co-localized with 28 rDNA sites.

### Painting FISH on *X*. *tropicalis* chromosomes

**Fig 3:**
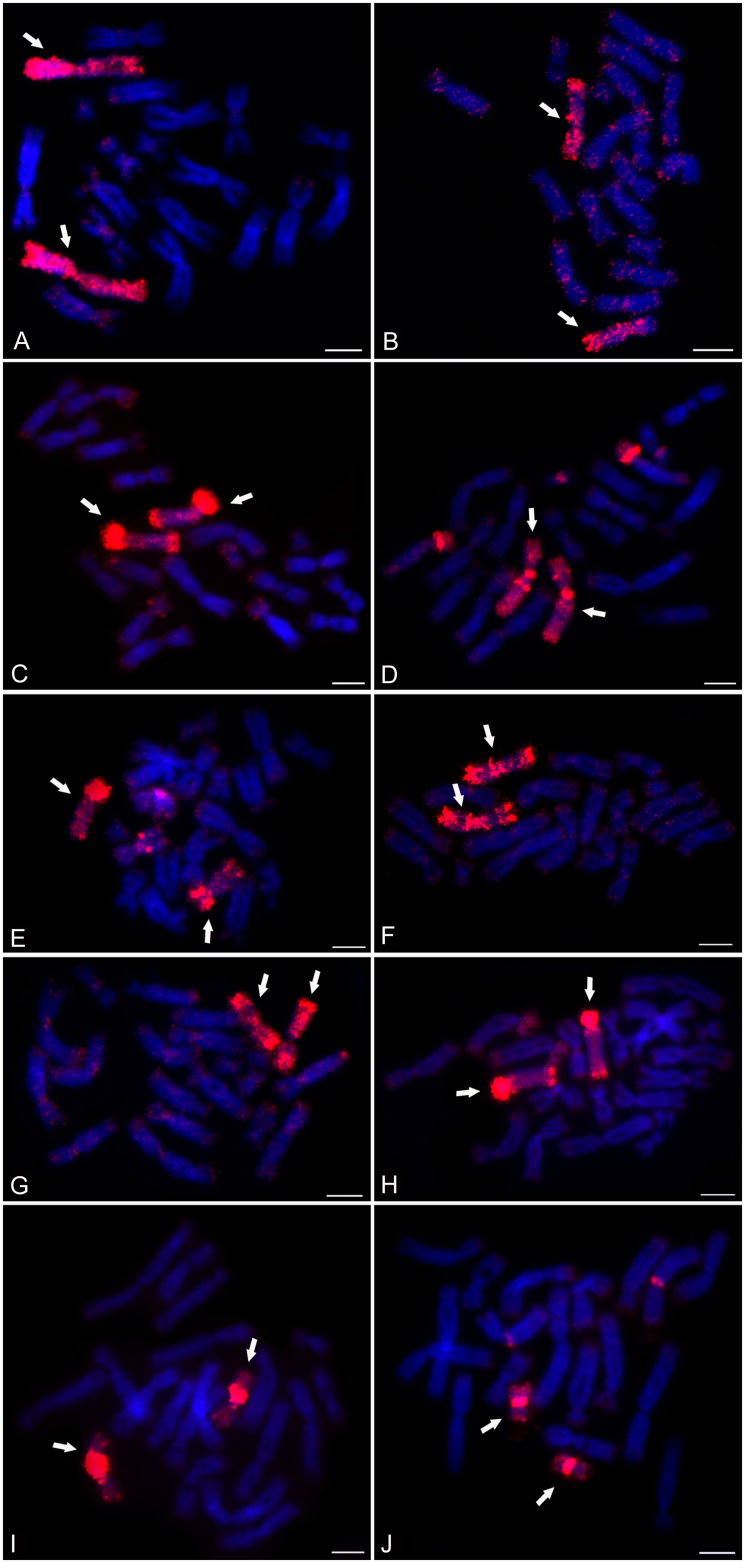
Painting FISH with *X*. *tropicalis* WCP probes. White arrows indicate staining of whole chromosome pairs using WCP probes derived from (A) XTR 1, (B) XTR 2, (C) XTR 3, (D) XTR 4, (E) XTR 5, (F) XTR 6, (G) XTR 7, (H) XTR 8, (I) XTR 9, and (J) XTR 10. In addition to red-stained homologous chromosome pairs, some WCP probes also had additional signal on telomeres and centromeres, which presumably is caused by high abundance of repetitive sequences. Scale bar represents 10 μm.

Ten WCP probes derived from XTR 1–10 were prepared from microdissected chromosomes by amplification using WGA4 kit (Sigma-Aldrich) and following reamplification by means of WGA3 kit (Sigma-Aldrich) including Dig-11-dUTP. As expected, each probe hybridized specifically and stained a whole chromosome pair (Fig 3).

### Zoo-FISH on *X*. *mellotropicalis* chromosomes

**Fig 4:**
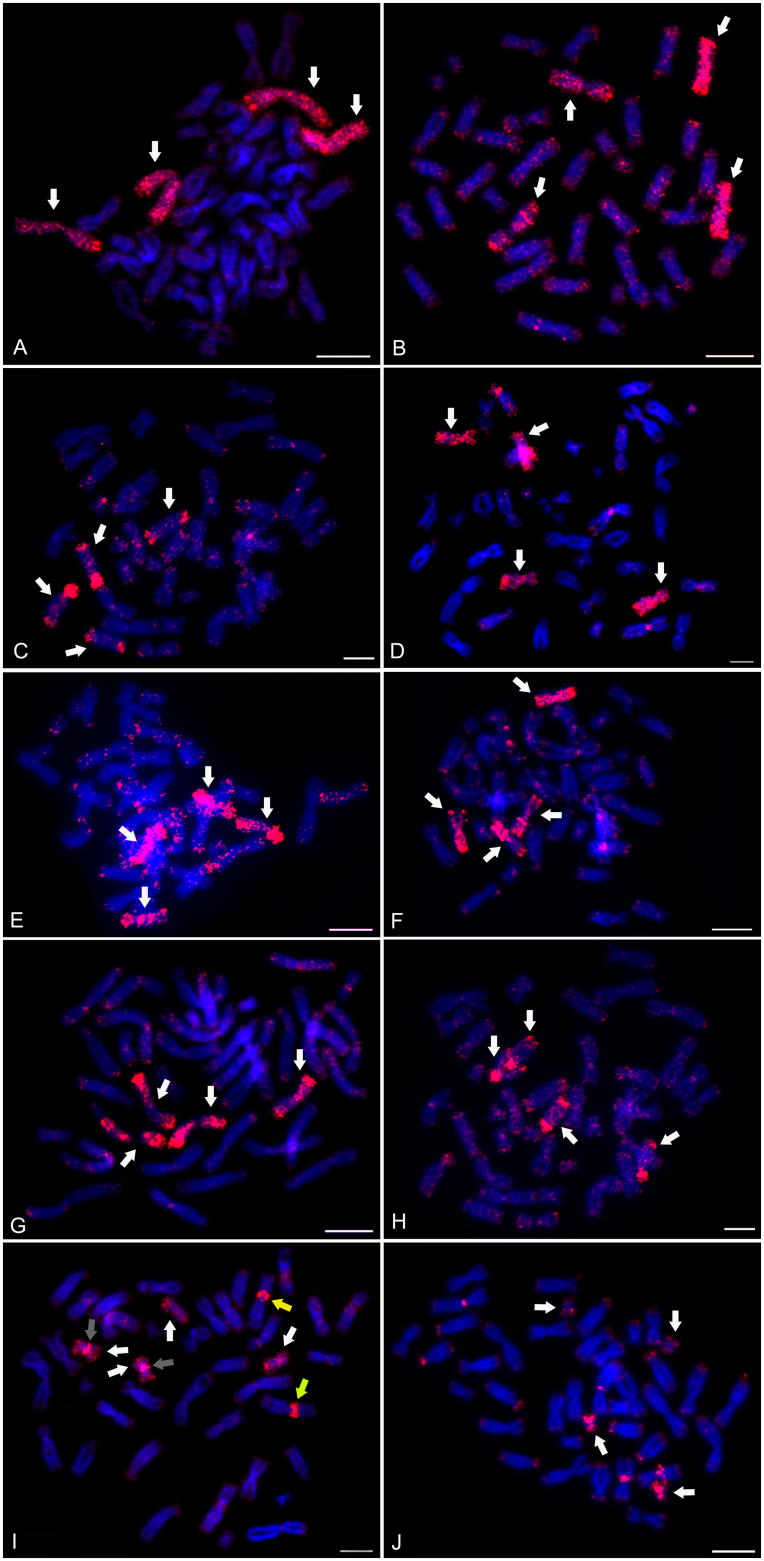
Zoo-FISH on *X*. *mellotropicalis* chromosomes using *X*. *tropicalis* WCP probes. XTR WCP probes stained the appropriate chromosomal quartets. (A) XTR 1 –XME 1a and 1b, (B) XTR 2 –XME 2a and 2b, (C) XTR 3 –XME 3a and 3b, (D) XTR 4 –XME 4a and 4b, (E) XTR 5 –XME 5a and 5b, (F) XTR 6 –XME 6a and 6b, (G) XTR 7 –XME 7a and 7b, (H) XTR 8 –XME 8a and 8b, (I) XTR 9 –XME 9a and 9b and (J) XTR 10 –XME 10a and 10b. White arrows show labelled chromosomal quartets. In (I) yellow arrows highlight the additional signals on XME 2a chromosome pair after hybridization with XTR 9 WCP. Gray arrows indicate the residue of constitutive heterochromatin in XME 9b. Scale bar represents 10 μm.

After confirming WCP specificity in *X*. *tropicalis*, we carried out Zoo-FISH experiments on *X*. *mellotropicalis* metaphase spreads using each *X*. *tropicalis* WCP probe. The WCP probes derived from XTR 1–10 hybridized to whole chromosomal quartets as follows: XTR 1 to XME 1a + 1b (Fig 4A); XTR 2 to XME 2a + 2b (Fig 4B); XTR 3 to XME 3a + 3b (Fig 4C); XTR 4 to XME 4a + 4b (Fig 4D); XTR 5 to XME 5a + 5b (Fig 4E); XTR 6 to XME 6a + 6b (Fig 4F); XTR 7 to XME 7a + 7b (Fig 4G); XTR 8 to XME 8a + 8b (Fig 4H); XTR 10 to XME 10a + 10b (Fig 4J). The XTR 9 probe revealed the whole chromosomal quartet XME 9a + 9b and also stained the *p* arm pericentric region on XME 2a (Fig 4I). The labelling of the chromosomes of *X*. *mellotropicalis* follows the convention used in Tymowska and Fischberg [13] where the larger of the homoeologous pairs is designed “a”and the smaller pair is designated with a “b“. Consequently, these designations do not reflect evolutionary affinities with respect to the chromosomes of *X*. *tropicalis*, in that some *X*. *mellotropicalis* chromosomes designated “a”might be more closely related to the orthologous chromosome in *X*. *tropicalis* than the *X*. *mellotropicalis* “b”chromosome, but the opposite might be true for another chromosome pair.

Moreover, for most of the homoeologous pairs of *X*. *mellotropicalis*, we were not able to distinguish which pair was most closely related to the orthologous chromosome pair of *X*. *tropicalis*, although there were some exceptions. In chromosomal quartets 2, 7, and 9, inferences of evolutionary affinities were possible: *X*. *mellotropicalis* chromosome pair 2b is more similar to *X*. *tropicalis* chromosome 2 based on size and Zoo-FISH. *X*. *mellotropicalis* chromosome pair 7a bears the secondary constriction found in XTR 7 and is more completely painted than *X*. *mellotropicalis* chromosome pair 7b, suggesting that *X*. *mellotropicalis* chromosome pair 7a is more closely related to XTR 7. For *X*. *mellotropicalis* chromosome pair 9b but not 9a we detected a small heterochromatic block, which we hypothesize to be a portion of a larger block that was present ancestrally but later translocated to chromosome 2a.

## Discussion

In this study we determined cytogenetic charactertistics of the Gabonese clawed frog, *Xenopus mellotropicalis*, a tetraploid species, and compared it to two close relatives: the diploid *X*. *tropicalis* and the tetraploid *X*. *epitropicalis*. Sequential fluorescent chromosome banding in *X*. *mellotropicalis* allowed identification of homologous chromosome pairs and chromosomal quartets comprised of homoeologous pairs of homologous pairs, as expected based on the tetraploid ancestry of this species. Comparison with previous cytogenetic analyses of *X*. *epitropicalis* [23] revealed similar banding patterns over most of the chromosomes of both of these tetraploid species, a result that is consistent with the close phylogenetic affinities inferred from DNA sequence data [17]. One key cytogenetic difference between these species was observed with respect to the chromosomal localizations of the constitutive heterochromatic block on XME 2a and XEP 2a. While in *X*. *tropicalis* this region is present on the *q* arm of XTR 9 [23], its position in both tetraploid species was found in a pericentric area of chromosomes 2a (*X*. *mellotropicalis—*this study, *X*. *epitropicalis* [23]). Zoo-FISH analysis employing XTR 9 WCP provided the evidence that this constitutive heterochromatic block was inserted (by non-reciprocal translocation or transposition) from the ancestral chromosome 9. While the positions of Zoo-FISH and C-banding regions overlapped in XME 2a, the small portion of this block was detected on *X*. *mellotropicalis* chromosomes 9b but not 9a (this study) and seems to be totally absent on both *X*. *epitropicalis* chromosomes 9 [23].

**Fig 5:**
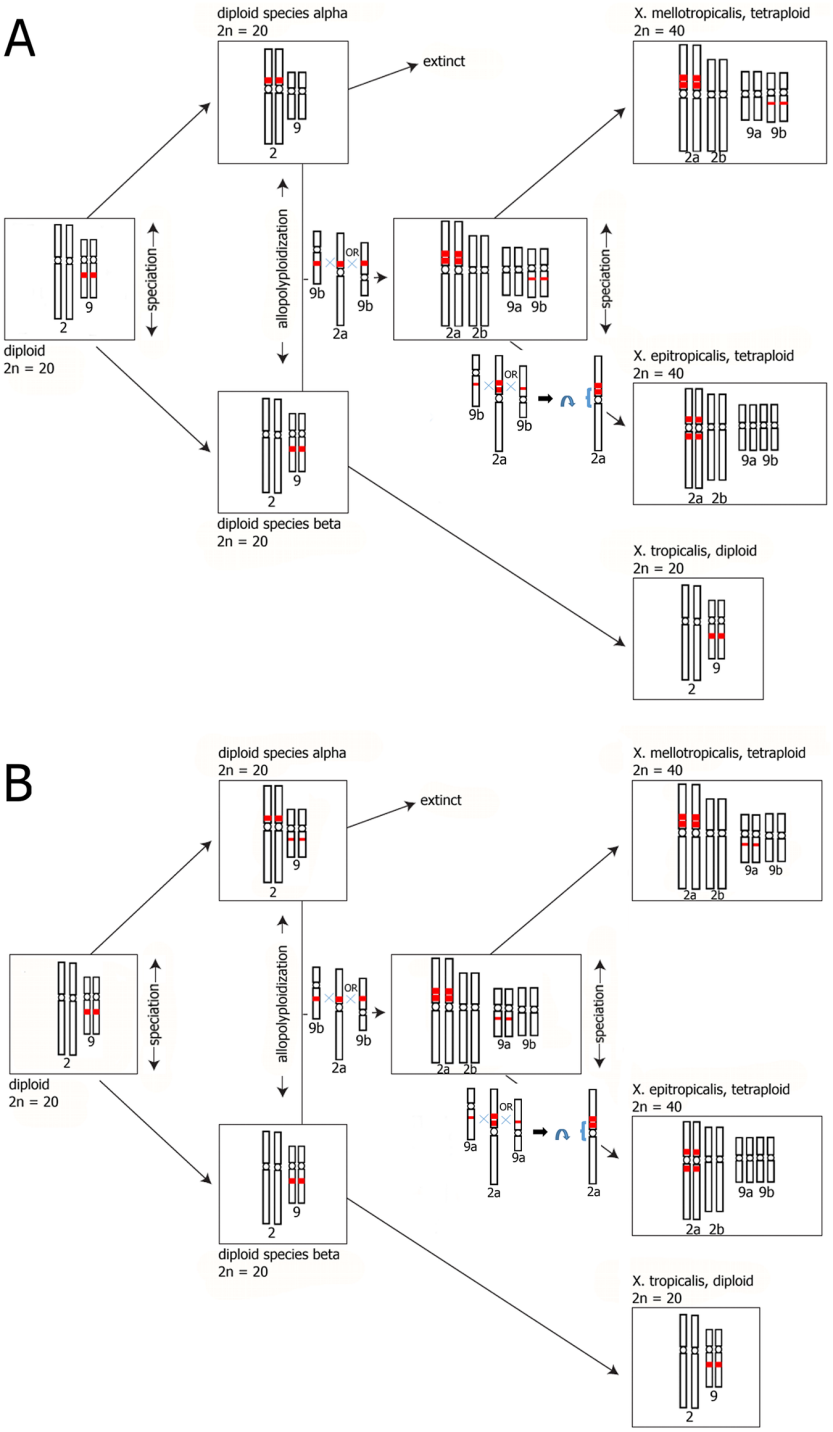
Potential evolutionary scenarios forming *X*. *mellotropicalis* and *X*. *epitropicalis* karyotypes based on a common allotetraploid ancestor. At least two evolutionary scenarios could form the allotetraploid species with the observed differences in the chromosomal locations of constitutive heterochromatic blocks. Scenario A indicates the translocation of a complete constitutive heterochromatic block from chromosome 9 to 2, and scenario B indicates the translocation of an incomplete constitutive heterochromatic block from chromosome 9 to 2. In (A), an ancestor of both allotetraploid species carried heterochromatic blocks on chromosomes 2a and 9b. In (B), genomic rearrangement produced a karyotype with the complete heterochromatic block on chromosomes 2a and 9b and a partial one on chromosome 9a. Sequence similarity of heterochromatic blocks from different chromosomes then facilitated a second incomplete (A) or complete (B) insertion of a heterochromatic block from chromosome 9b to 2a, which gave rise to the *X*. *mellotropicalis* karyotype. A residual hetrochromatic block on *X*. *mellotropicalis* chromosomes 9 was revealed by Zoo-FISH and is thus located either on chromosome 9b (A) or chromosome 9a (B). The formation of the *X*. *epitropicalis* karyotype from an allotetraploid ancestral species could have occurred via complete non-reciprocal recombination of a heterochromatic block between chromosomes 9a (or 9b) and 2a followed by an asymmetric pericentric inversion on chromosome 2a. Chromosome labelling “a”and “b”reflects the ancestral karyotypes (i.e. from which diploid ancestor a homoeologous pair is derived) and does not necessarily correspond with the chromosome names in the text which are based on the relative size of each homoeologous pair [23]. Chromosomal rearrangements between hypothetical or observed karyotypes of ancestral and extant species depicted out of the rectangular boxes are represented on haploid chromosomes.

In African clawed frogs of the genus *Xenopus*, speciation typically occurs where one ancestral species diverges into two descendant species. However, allopolyploidization has also occurred several times, wherein new polyploid species are generated in association with hybridization among species [17]. In *X*. *mellotropicalis*, only two of the four homoeologous chromosomes 2 (2a) contain a constitutive heterochromatic block. This result could be explained by the existence of two diploid ancestors differing in the chromosomal location of this constitutive heterochromatic block. The first diploid ancestor may have resembled extant *X*. *tropicalis* in that this region was located on chromosome 9. In the second diploid ancestor, which is probably now extinct, this constitutive heterochromatic block was located on the *p* arm in the pericentric region of chromosome 2. Allotetraploidization then gave rise to a tetraploid ancestor of *X*. *mellotropicalis* + *X*. *epitropicalis* that had a duplicated heterochromatic block on chromosomes 2a and 9b (Fig 5). Thus, under this scenario, the different locations of the constitutive heterochromatin on chromosomes 2a and 9b would be due to translocation in one of the diploid species after divergence from a common ancestor. As such, we would expect high DNA sequence identity between these blocks, which could promote pairing and further reciprocal or non-reciprocal translocation. Compared to *X*. *tropicalis* [36] and *X*. *epitropicalis* [23], our morphometric analysis recovered a substantial difference in the *p*/*q* arm ratio for *X*. *mellotropicalis* chromosome 2a. The *p* arm is longer, and the XME 2a chromosome pair is almost metacentric, whereas XME 2b is submetacentric. In addition, comparison of morphometric values of XTR 9 [36] with XME 9a and b (this study) and XEP 9a and b [23] indicates that the *q* arms of both homoeologous chromosome 9 pairs in both tetraploid species are considerably shorter than the orthologous XTR 9. Consequently, the *p*/*q* arm ratio in XTR 9 corresponds to submetacentric and in XME 9a, b and XEP 9a, b is almost metacentric (Table 1). We interpret these results to suggest either that (1) a deletion occurred on both homoeologous chromosome 9 pairs in an ancestor of both of these tetraploids or that (2) an insertion occurred on XTR 9. Zoo-FISH analysis employing XTR 9 WCP revealed a residue of heterochromatic block on XME 9b. We thus speculate that a non-reciprocal incomplete translocation of heterochromatic block from chromosome 9b to the *p* arm pericentric region of chromosome 2a occurred either in the allotetraploid predecessor of *X*. *mellotropicalis* + *X*. *epitropicalis* (Fig 5, scenario A) or in one of the diploid ancestors of these tetraploids (Fig 5, scenario B). This translocation could have initiated a cytogenetic difference between both tetraploids and *X*. *tropicalis*. In *X*. *epitropicalis*, the constitutive heterochromatin occurs on both arms of XEP 2a pericentric area [23], whereas in *X*. *mellotropicalis* there are two bands of constitutive heterochromatin on only the *p* arm of XME 2a. Since the *p*/*q* arm ratio of *X*. *epitropicalis* chromosome 2a is similar to XTR 2 (Table 1), it is possible that an asymmetric pericentric inversion occurred in *X*. *epitropicalis* after divergence from *X*. *mellotropicalis*, and that the distribution of heterochromatic blocks on XME 2a resembles the allotetraploid ancestral state. It seems that the residue of heterochromatic block detected in XME 9b was lost or completelly non-reciprocally translocated on 2a chromosomes in *X*. *epitropicalis* (Fig 5). This is evidenced by an absence of substantial heterochromatic block on XEP 9b and the same *p*/*q* arm ratio between XEP 9a and 9b (0,80) [23], which is not true for *X*. *mellotropicalis* homoeologs (9a = 0,88, 9b = 0,72).

Our results indicate that at least two of the three tetraploid species in the subgenus *Silurana* (*X*. *mellotropicalis* and *X*. *epitropicalis*; high resolution cytogenetic data are lacking for *X*. *calcaratus*) originated from one allotetraploidization event. Previous data based on the sequencing of mitochondrial genes [21] and on tightly linked nuclear genes *RAG-1* and *RAG-2* derived from both homoeologous chromosomes [30,31] have also suggested this evolutionary scenario. The similar evolutionary scheme was also previously proposed to have generated the most recent common ancestor of all species (including *X*. *laevis*) with 36, 72, or 108 chromosomes, which together comprise the subgenus *Xenopus* [20,22,27]. Fusion of the orthologs to XTR 9 and XTR 10 probably occurred in the diploid ancestor of this ancestral allotetraploid of the subgenus *Xenopus* before allotetraploidization [20,28]. Whole genome sequencing of *X*. *laevis* [28] identified substantially higher frequency of intra-chromosomal rearrangements, inversions, deletions or tandem duplications in one subgenome (the “S”subgenome) as compared to the other subgenome (the”L”subgenome). Thus, in this species, one subgenome was structurally more stable during evolution than the other. Zoo-FISH analysis of the *X*. *mellotropicalis* karyotype revealed an interchromosomal non-reciprocal translocation of heterochromatic block between 9b and 2a in a common allotetraploid predecessor. Because we were unable to determine whether *X*. *mellotropicalis* 9a or *X*. *mellotropicalis* 9b is more closely related to *X*. *tropicalis* chromosome 9, at this time we cannot determine whether the chromosomal instability we detected affected one or both subgenomes of *X*. *mellotropicalis*. An improved understanding of the above alternatives migth be provided by studying *X*. *calcaratus* (2*n* = 4*x* = 40) another relative species to both allotetraploids. Interchromosomal rearrangements between chromosomes derived from different subgenomes were described in *X*. *borealis* individuals affecting the number of NORs from one to three [25].

Concerning the FISH analysis with 28S rDNA probe, nucleolar constrictions with NORs were found on XTR 7 (Fig 2B) and XME 7a (Fig 2E). In general, NORs represent chromosomal regions with higher mutation rate [1,25]. Their mutability (e.g. chromosomal deletion) is associated with the reduction of NOR number after a polyploidization event [40], which may be the reason why only one NOR pair is present in polyploid *Xenopus* species. Variation in the number, position, morphology and function of two basic constriction types (non-specific and nucleolar) could be used for species determination in the subgenera *Xenopus* or *Silurana* with the same chromosome numbers [16]. 5S rDNA loci were situated in telomeric region of both chromosomal arms in *X*. *tropicalis* and *X*. *mellotropicalis*. Suprisingly, their number in diploid *X*. *tropicalis* was higher (eight from ten chromosome pairs) than in allotetraploid *X*. *mellotropicalis* (three from 20 chromosome pairs) (Fig 2C and 2F). Variation in the number and position of 5S rDNA hybridization sites have been also observed in *X*. *laevis*, *X*. *muelleri* [41] and *X*. *borealis* [22,42]. Both Schmid and Steinlein [22] and Pardue [42] found 5S rDNA loci only on telomeric chromosomal segments. In addition, Schmid and Steinlein [22] identified a high number of 5S rDNA sequences on the *X*. *laevis* and *X*. *borealis q* arms but only one chromosome pair carrying 5S rDNA site in *X*. *muelleri*. Our results corroborate the postulated exceptionally high number of 5S rDNA loci found in *X*. *laevis* and *X*. *borealis* among amphibians, supporting rather high variability in the number of 5S rDNA loci in this class [22].
